# Suicide risk assessment: clinical implications of the unpredictability of suicidal behavior

**DOI:** 10.3389/fpsyt.2026.1844322

**Published:** 2026-06-02

**Authors:** Tobias Teismann, Wilco C. Janssen, Henriëtte D. Heering

**Affiliations:** 1 Mental Health Research and Treatment Center, Faculty of Psychology, Ruhr-Universität Bochum, Bochum, Germany; 2 DZPG (German Center for Mental Health), Bochum, Germany; 3 113 Suicide Prevention, Amsterdam, Netherlands; 4 Department of Psychiatry, Amsterdam UMC location AMC, Amsterdam, Netherlands; 5 GGZinGeest Specialized Mental Health Care, Amsterdam, Netherlands

**Keywords:** needs-based approach, risk assessment, risk-stratification, severity-based approach, suicide

## Abstract

Risk assessment in the context of suicidal ideation and behavior cannot be conducted with certainty. Recent meta-analyses demonstrate that neither individual risk factors, composite risk scores, clinical judgment, nor adherence to theoretical models or artificial intelligence enables sufficiently accurate prediction of suicidal behavior. This raises the question of how clinicians should respond to the well-documented limitations in the precision of risk assessment. This article first reviews the current state of empirical evidence and subsequently reflects on the implications of these findings for clinical practice. Three alternative approaches to risk assessment are presented. Building on these, an integrated model for risk assessment is introduced in a practice-oriented manner.

## 1 Introduction

In various clinical and institutional settings, there is an obligation to initiate, conduct and document a suicide risk assessment for individuals suspected of experiencing suicidal ideation. Screening tools are available to gain an initial impression and there are guidelines and manuals that specify which risk and protective factors should be assessed in detail (1). In addition, there are instructions explaining the conditions under which a low, moderate, high, or extreme suicide risk can be assumed (2, 3). Decades of epidemiological and clinical research have indeed established a reasonably consistent inventory of variables associated with elevated suicide risk at the *group* level. These include psychiatric diagnoses, prior suicide attempts, psychological pain and hopelessness, childhood adversity, impulsivity, social disconnection, access to lethal means, and neurobiological markers [e.g., (4–8)]. Indeed, such publications easily give the impression that a person’s suicide risk can be assessed objectively, reliably, and validly. At the same time, there are substantial doubts as to whether this is actually feasible.

The current paper pursues three interrelated aims: first, to summarize why prediction-based approaches to suicide risk assessment lack clinical reliability at the level of the individual patient; second, to argue that this limitation is structural rather than merely technical, and thus unlikely to be resolved through incremental methodological refinement; and third, to propose an integrated clinical alternative grounded in patient needs, current suicidal phenomenology, and decision-making capacity. The present article advances a narrative synthesis of meta-analyses, clinical guidelines, and influential longitudinal studies on suicide prediction and the clinical assessment of suicidality, with studies selected for their conceptual relevance, recency, and scholarly influence rather than through a formal systematic review protocol.

## 2 Empirical findings

Various studies have demonstrated that the reliability of risk assessments is moderate at best (9), and that the prediction of suicidal behavior, is currently insufficient for clinically useful individual-level prediction (10). The latter has been repeatedly pointed out since the 1950s (11–16), but so far without widespread resonance in clinical practice and parts of the research world. Some milestone works on this topic are summarized below.

Franklin and colleagues (10), for example, conducted a widely cited meta-analysis to investigate which factors are particularly important for predicting suicide attempts and suicides. The authors considered a total of 365 longitudinal studies and 16 types of risk factors, including demographic characteristics, psychopathology, personality factors, history of suicidal ideation and behavior, medical history, and biomarkers. Despite the extensive data material and the diverse analyses, the results showed that (a.) the prediction of suicide attempts and suicides has not improved in the past 50 years, despite an immense increase in research in this area and that (b.) when considering any risk factor, prediction is only marginally better than random chance. At the same time, the meta-analysis has limited significance for clinical practice: no therapist would focus in a risk assessment on the presence of one or two individual factors, but rather on a complex clinical impression. A combination of many risk factors and their interaction with potential protective factors is therefore used to assess risk.

However, a meta-analysis by Large and colleagues (17) showed that even when a large number of different sources of information are combined to categorize patients as high-risk vs. low-risk, the predictive power for suicidal behavior is still insufficient. This meta-analysis included 37 longitudinal studies of psychiatric patients in which at least two variables were combined to identify a high-risk group. A statistically highly significant association was found between belonging to the high-risk group and suicide: during an average follow-up period of 63 months, 5.5% of high-risk patients died by suicide compared to 0.9% of patients who were not assigned to the high-risk group. At the same time, however, this implies that 95% of those classified as high risk did *not* die by suicide and sensitivity analyses indicated that 44% of the patients who died by suicide were *not* assigned to the high-risk group. In another meta-analysis, Woodford and colleagues (18) investigated the extent to which clinicians can predict which of their patients will engage in self-injurious behavior after being discharged from inpatient treatment. It was found that 70% of those who engaged in self-injurious behavior after treatment had previously been assessed by their practitioners as not being at risk and that around 80% of those who were assessed as being at high risk did not engage in self-injurious behavior. Taken together, the predictions made by practitioners in this meta-analysis were only slightly better than chance. It therefore does not appear to be possible for practitioners to make meaningful predictions about self-injurious behavior, suicide attempts or suicides.

### 2.1 Attempts to improve suicide risk assessment

This raises the question of whether risk assessment can be improved, for instance if practitioners use theoretical models of suicidal ideation and behavior as a guide. After all, this approach should focus attention on factors that are particularly important for understanding suicidal ideation and behavior.

Meta-analyses of two important theoretical models, the Interpersonal Theory of Suicidal Behavior (19) and the Integrative Motivational-Volitional Model of Suicidal Behavior (20, 21), show results that are consistent with the theories. The effects are, however, comparatively small (22, 23) and do not exceed the predictive power found in the other meta-analyses described above. Theoretical models, in other words, do not appear to offer a meaningful improvement in the prediction of suicidal behavior (however, see also (24).

Recently, there has been hope that the prediction of suicidal behavior can be improved through the use of artificial intelligence (AI). One advantage of AI over conventional statistical approaches is that thousands of influencing factors and their interactions can be taken into account simultaneously when predicting events of interest. Another advantage of AI is that it can be used as a self-learning system, i.e., it is not determined *a priori* which variables are to be taken into account and in what way. Rather, the algorithm iteratively derives the criteria best suited for classification. Initial results indicated a comparatively high accuracy of risk classification in predicting suicidal ideation and behavior [e.g., (25)] but expectations regarding the possibilities of suicide prediction using AI models have since been diminished: Even complex risk assessment models do not allow for precise prediction of suicidal behavior (26, 27) and, in particular, they do not allow for prediction of the time at which suicidal behavior will occur.

It should therefore be noted that neither individual variables nor clinical judgment, orientation toward a theoretical model, or artificial intelligence allow for clinically useful individual-level prediction. The same also applies to suicidal syndromes (28) and implicit measures for assessing suicidal intentions (29).

### 2.2 The effect of time horizon

To make matters worse, the figures cited above mainly concern predictions over periods of months to years. In clinical practice, however, practitioners are typically asked to assess whether a patient is at risk of suicide within the next hours, days, or weeks, for example, to decide whether hospitalization is warranted. Paradoxically, the shorter the time horizon, the worse the predictive value of risk assessments becomes. This is a direct mathematical consequence of base rates: the absolute probability of suicide decreases as the time window shrinks, simply because less time has elapsed. A patient who will die by suicide three months from now is still alive after one week, meaning that any high-risk classification is a false positive at that point. **Table 1** illustrates the potential magnitude of this effect. The figures shown are not empirical results, but a modeled example based on published post-discharge suicide rates (30, 31) combined with the sensitivity and specificity estimates reported by Large et al. (17); the underlying calculations are summarized in the table note. The pattern is nonetheless striking: even in the post-discharge period, one of the highest-risk contexts in clinical psychiatry, the ratio of false positives to correct predictions increases from approximately 77 per correct prediction over one year to an estimated 4,600 or more over a single day. In populations with a lower base rate of suicide, which includes the majority of clinical settings, these ratios would be considerably worse. It should be noted, however, that the limits described here apply to individual-level prediction. They do not preclude the possibility that biomarker- or machine-learning-based approaches may contribute incrementally at the population level - for example, by identifying higher-risk subgroups for targeted preventive intervention or by informing resource allocation across health systems. The clinical limitation lies in translating such population-level signal into reliable predictions about specific individuals at specific moments in time.

**Table 1:** Estimated false positives per correct prediction of suicide at different time horizons, in discharged psychiatric inpatients.

Time window	Suicide rate¹	Base rate	PPV²	False positives per correct prediction
1 day	~2,950/100k/yr†	0.008%	0.02%	~4,600
1 week	2,950/100k/yr	0.06%	0.15%	~660
1 month	2,060/100k/yr	0.17%	0.45%	~220
3 months	1,132/100k/yr	0.28%	0.74%	~135
1 year	484/100k/yr	0.48%	1.28%	~77

Modeled illustrative example, not an empirical result of the present study. PPV was calculated using Bayes’ theorem: PPV = (sensitivity × base rate)/[(sensitivity × base rate) + (1 − specificity) × (1 − base rate)], with sensitivity = 56% and specificity = 79% from Large et al. (2016). False positives per correct prediction = (1 − PPV)/PPV.

¹Empirically measured pooled rates from Chung et al. (2017, 2019); rates decline non-linearly after discharge.

²PPV at the indicated time horizon, derived as above.

†No empirical daily rate available; the first-week rate is used as a coarse proxy. The actual daily rate likely varies across the first week, so this row should be read as an order-of-magnitude illustration rather than a precise estimate. The proxy nonetheless overestimates the daily base rate on average, making the false positive ratio a conservative estimate.

### 2.3 Reasons for the lack of predictability of suicidal behavior

The principal reason for the limited ability to predict suicide deaths and attempts with reliability is the low base rate of suicide. Bryan (11) illustrates this with a model calculation based on a study in which more than 80,000 people were examined over a period of four years (32). At the start of the study, all participants completed the Patient Health Questionnaire (PHQ-9), in which they were asked whether they had had thoughts of wanting to be dead or harming themselves in the past two weeks. There were four possible answers: not at all, on some days, on more than half of the days, or almost every day. Participants who reported having suicidal thoughts almost every day in this questionnaire had a tenfold higher risk of dying by suicide or attempting suicide during the study period than participants who reported not having thought about suicide at all. A tenfold increase in suicide risk sounds as if there is indeed a high risk of suicide. However, a closer look at the figures reveals that this is by no means the case. Of the people who reported not thinking about suicide at all, 1 in 3,000 died by suicide during the study period. Of those who reported thinking about suicide almost every day, ten times as many people died by suicide during the study period, i.e., 10 people per 3,000. In the group of people without suicidal thoughts, the risk of suicide was 0.03%, and in the group of people with daily suicidal thoughts, the risk of suicide was 0.3%. Of course, this is a significantly increased statistical risk. At the same time, however, this does not mean that there is a high risk, because 99.7% of people with daily suicidal thoughts do not die by suicide! Accordingly, a factor may show a strong relative association with suicide while still performing poorly in clinically meaningful prediction of individual outcomes. Consequently, as the meta-analyses discussed above indicate, knowledge of statistically significant risk factors does not translate into reliable or clinically useful predictions of suicide. “To put this into perspective, if we designed a nearly perfect suicide-risk screening scale that had 99% sensitivity (i.e. identified 99 out of 100 suicide decedents) and 99% specificity (i.e. identifies 99 out of 100 nonsuicide decedents) and then used it in a group of people with a suicide rate of 20,000 per 100,000, its positive predictive value would be 96%, meaning a positive screen on the scale would almost always be right. If that same, nearly perfect scale were used to screen everyone in the United States, however, which has a much lower prevalence rate of 13 per 100,000, the positive predictive value would be 1.3%, meaning that a positive screen would almost always be wrong [11, p (68)]. In conclusion, prediction of rare events is not accurate enough to justify high-stakes stratification decisions.

The main reason why it is impossible to predict suicides is therefore the low base rate of suicidal behavior. However, suicide risk assessment is further complicated by other factors:

- In approximately 50 to 60% of those affected, suicidal ideation and behavior are not disclosed to others (33) and therefore cannot be identified as at risk.
- Suicidal individuals typically experience suicidal ambivalence (34) and suicidal ideation is subject to considerable fluctuations in intensity (35), with potentially rapid transitions from a decision to die by suicide and suicidal actions (36). A suicide risk assessment carried out at a specific point in time therefore often has only a very limited temporal validity.
- Suicidal behavior does not represent the end point of a uniform development: In this sense, 11-17% of respondents report that neither suicidal thoughts nor a suicide plan preceded their suicide attempt (37, 38).

All of the above points explain why suicide risk assessment does not allow for the reliable identification of individuals with a particularly high risk of suicide or individuals with a particularly low risk of suicide.

### 2.4 The risks of risk assessment

Given this evidence, the routine use of suicide risk assessment in clinical practice carries real costs that are easily overlooked: (1.) When a patient is classified as low risk, little is typically done to prevent suicide, even though, as the figures above show, a substantial proportion of those who do die by suicide will have received exactly this classification. In the worst case, the patient does not receive access to evidence-based treatment, or there is no, or only very limited, follow-up care after an inpatient admission. (2.) When a patient is classified as high risk, the consequences can be far-reaching: trauma treatment may be postponed, freedom of movement restricted, and access to parts of the mental health system denied, even though approximately 95% of those classified as high risk will never die by suicide. By conventional standards, a false positive rate of this magnitude would disqualify a test from informing major clinical decisions. Beyond its limited validity, risk assessment also carries relational and institutional costs.

On this background, international clinical guidelines are increasingly cautioning against prediction-based risk stratification, as illustrated by the English NICE Guideline on the treatment of self-harm (39), which issues the following warnings, among others:

- Do not use global risk stratification into low, medium, or high risk to predict future suicide or repetition of self-harm.
- Do not use global risk stratification into low, medium, or high risk to determine who should be offered treatment or who should be discharged [cf. (40)].

## 3 Implications for risk assessment in clinical practice

In clinical practice, the question now arises as to how to deal with the knowledge of the substantial difficulties involved in risk assessment. Ryan and colleagues (40), p. 402) put it very clearly: “… risk categorization has no useful role to play as a guide to clinical decision-making and should be abandoned.” However, this does not imply that suicide-focused conversations should be abandoned. There are at least three reasons for continuing such conversations:

1. From a legal perspective, a structured exploration of suicidal ideation/behavior and the factors influencing it is a standard of care in many countries, and failure to do so could have legal consequences in the event of a suicide [e.g., (41–43)]. Just to be clear: The legal standard does typically not require clinicians to *predict* suicide, but to assess carefully and act reasonably on what was knowable at the time.
2. From a patient perspective, it should be emphasized that many suicidal patients express a desire for someone to talk to them in detail about their suicidal thoughts (44). A suicide-focused conversation therefore often fulfills a patient’s wish.
3. From a therapeutic perspective, the suicide-focused conversations are important to gather information on the basis of which a case concept can be developed and a therapy plan can be drawn up.

Several alternatives to the risk-based approach to suicide prevention have been proposed.

### 3.1 The needs-based approach to suicidality assessment

First, the *needs-based approach* holds that conversations about suicidality should focus not on risk estimation but on identifying modifiable factors that bring someone closer to or further from a suicide attempt (13), thereby informing the selection of treatment targets. Several concrete implementations of this approach have been proposed. The first is the narrative interview, in which patients are invited to describe openly how their suicidal crisis or suicide attempt came about (45, 46). An initial study found that a narrative interview was associated with a greater reduction in suicidal ideation than a standardized risk assessment (47), and a recent meta-analysis found that CBT interventions including a narrative assessment were associated with a significantly reduced risk of suicide attempt compared to controls, whereas CBT without this component was not (48). The second, proposed by Teismann and colleagues (49, 50), retains the structure of a conventional risk assessment interview but shifts its purpose. Rather than gathering information to estimate risk, the clinician aims to understand the nature and background of the patient’s suicidality - what caused it, what maintains it, and what the patient needs in order to feel safer. In this sense, an interview with a suicidal patient is no different from an interview with, for example, a depressed patient. Here, too, therapists want to understand how the depression developed, what is maintaining depressive symptoms, and what patients want from treatment. The aim is therefore to develop an understanding of the case and identify patient goals, not to predict how depressed the patient will be in the future. A third approach is the CAMS framework proposed by Jobes (51). Within this model, a collaborative treatment plan is developed on the basis of a structured interview in which patient and clinician jointly explore the individual “drivers” of suicidality as well as the patient’s reasons for living and for dying. The CAMS approach has been examined in a number of studies and has demonstrated effectiveness in reducing suicidal ideation (52); however, its efficacy in preventing suicide attempts remains to be established in future research.

The needs-based approach sidesteps the unpredictability of suicide by not requiring prediction at all. Instead, it focuses on understanding what is driving and maintaining the current crisis, directly informing clinical action. A drawback is that the approach offers guidance on what to do, but is less clear about when or with whom. That is, it provides no explicit decision rule about when preventive action is warranted. Also, it may take somewhat more time, although a narrative assessments can usually be completed in approximately 10 minutes (45).

### 3.2 The severity-based approach to suicidality assessment

The second proposed alternative to risk assessment is the *severity-based approach*. Although the assessment of severity may initially appear similar to estimating risk, the two differ in fundamental ways. Suicidal thoughts and behaviors are conceptualized not only as predictors but also as precursors to suicide, and the focus shifts from forecasting future outcomes to describing the current clinical presentation. Whereas risk assessment seeks to predict how severe suicidality may become, severity-based assessment focuses on the suicidal thoughts, feelings, and behaviors that are already present or have occurred; as such, it entails a detailed exploration of the phenomenology of suicidal ideation, including its frequency, duration and persistence, intensity, variability, controllability as well as suicide intent, planning, recent suicidal escalation as well as proximity to action. This information is then used to inform treatment planning via what Obegi (53) describes as the precautionary principle: when a threat of harm is plausible and serious but cannot be meaningfully quantified, action is justified not by a prediction but by a value judgment - that inaction in the face of potential harm to human life is unacceptable. The principle is familiar from somatic medicine, where chest pain warrants graded assessment and intervention based on the severity of the presentation rather than on a probabilistic estimate of who will die. Translated to suicidality, severity does not reliably predict who will die by suicide, but it does describe a clinically credible threat - and the principle that “the greater the danger, the greater the care” links severity to proportional intervention without requiring accurate prediction. For example, the severity and chronicity of suicidality may inform decisions regarding whether, and to what extent, a combined pharmacological and psychotherapeutic treatment is likely to be beneficial [cf. (54)]. This reasoning parallels established practices in the treatment of depression, where therapeutic approaches are similarly differentiated according to the temporal course (chronic versus acute) and severity (mild versus severe) of the condition (55).

In conclusion, the severity based approach is less time-consuming than a risk assessment, since only suicidality itself needs to be evaluated rather than a broad range of associated risk factors. One structured operationalization of this approach is the Chronological Assessment of Suicide Events (CASE (56);, which systematically maps the timeline and content of a patient’s suicidal ideation and behavior. That said, the severity-based approach does not fully escape the underlying statistical problem. Most patients assessed as severely suicidal will not die by suicide in the near term, while a proportion of those who do will have presented as less severe. Assigning interventions with serious downsides such as involuntary hospitalization, or withholding effective ones without such downsides on the basis of severity alone raises similar ethical concerns as risk-based decision making. The philosophical basis may be more defensible, but the practical concerns remain. Also, assessing severity without identifying what drives this suicidality does not teach patients and clinicians much about what to do next. In this sense, the problem of the severity-based approach is exactly the opposite of that of the needs-based approach, which also makes them complementary.

### 3.3 The capacity based approach to suicidality assessment

A third alternative, proposed by Large and Ryan (57) suggests that clinicians should assess whether a patient has the capacity to make their own decisions about their health and safety. This ‘capacity based approach’ is a clinical-ethical framework, that implies that coercive intervention is only justified when a patient lacks decision-making capacity, not when their suicide risk is judged to be high. The assessment of mental capacity in suicidal patients encompasses several core components: First, understanding refers to the individual’s ability to comprehend information relevant to decisions about their safety and care, including the nature of their current crisis, available treatment options, and the associated risks and benefits. Second, appreciation denotes the capacity to apply this information to one’s own situation, that is, to recognize the personal significance of their suicidality, including the potential consequences of their decisions for their own safety and well-being. Third, reasoning involves the ability to weigh different courses of action - such as accepting or refusing treatment - and to provide a coherent and plausible rationale for these choices. Finally, expressing a choice refers to the ability to communicate a clear and consistent decision regarding treatment and safety (58). Patients who are deemed competent to make their own decisions should have those decisions respected, even if they carry risk. This approach has the advantage of offering the type of decision rule the needs-based approach lacks: coercive intervention is warranted when, and only when, a patient’s decision-making capacity is impaired. Its main limitation is that capacity assessment in suicidal patients is itself complex and contested (59). Cognitive and affective features that often accompany serious suicidality such as hopelessness, tunnel vision, and extreme psychological pain may be relevant to judgments about decision-making capacity. In practice, this makes it difficult to draw a clear line between impaired and intact autonomy. In this sense there is evidence suggesting that (facets of) mental capacity may be impaired in quite a few suicidal patients, meaning that clinicians may need to assume greater responsibility for these patients and their care (60). Finally, it is worth noting that, like the severity-based approach, this proposal rests not only on empirical grounds (i.e. the poor validity of risk assessment) but also on an explicit value judgment: Autonomy is a paramount right and the burden of proof for overriding it lies with those who would intervene, not with the patient.

A comparison of the three different approaches is presented in **Table 2**.

**Table 2:** Comparison of alternative approaches to suicidality risk assessment.

Category	Needs-based approach	Severity-based approach	Capacity-based approach
Primary aim	Identify modifiable drivers of suicidality and inform treatment targets	Describe current suicidal thoughts and behaviors	Determine whether a patient has capacity for autonomous decision-making
Core clinical questions	What caused and maintains suicidal ideation/behavior? What does the patient need to feel safer?	What suicidal thoughts, feelings, and behaviors are currently present?	Does the patient have sufficient mental capacity to make informed decisions about their safety?
Likely outputs	Narrative understanding of the suicidal crisis; identified therapeutic targets and patient goals	Structured severity profile of suicidality (e.g., timeline and intensity)	Mental capacity judgment (intact vs. impaired)
Strengths	Action-oriented; directly informs treatment; avoids prediction problem; clinically intuitive	Avoids prediction problem; clearer description of current state	Provides decision rule tied to autonomy; ethically grounded in respecting patient choice
Limitations	No clear threshold for intervention; may require more time; unclear “when” decisions should be made	Does not “explain” underlying causes (i.e. suicide drivers)	Capacity assessment is complex and contested in suicidal patients; boundaries between impaired and intact capacity are difficult to define
Implications for decision-making	Informs treatment planning but leaves intervention thresholds unspecified	Informs treatment planning regarding treatment setting and dosage, but leaves intervention focus unspecified.	Coercive intervention justified only when capacity is impaired; otherwise patient autonomy should be respected

### 3.4 A proposed comprehensive approach to suicide risk assessment and treatment planning

The various approaches may also be integrated within a single clinical encounter. In this sense, an assessment with a suicidal patient may begin with a narrative interview [e.g., “When were you closest to suicide in the past few weeks?” … “Tell me the story of that moment exactly as you experienced it — starting where you feel it began.” (46)]. This can then be complemented by a more fine-grained exploration of the characteristics of current suicidal ideation [cf. (56)], an examination of the underlying psychological processes or “suicide drivers” (51, 61), and a clarification of the patient’s goals and expectations with regard to crisis intervention and/or treatment. In this way, the needs-based approach - encompassing a narrative interview, the identification of drivers of suicidality, and the clarification of patients’ support needs - is integrated with a severity-based approach, which entails a detailed exploration of the phenomenology of suicidal ideation, including its intensity, chronicity, and variability. Like the needs-based approach, this latter perspective is not intended to predict risk; rather, it characterizes the severity of the current presentation to inform the proportionality of the clinical response. In this sense, the needs-based approach - beyond fostering a collaborative therapeutic relationship - supports the identification of treatment priorities and the formulation of specific therapeutic goals. By contrast, the severity-based approach provides, among other things, guidance regarding treatment intensity and, potentially, the appropriate treatment setting (see above). Consistent with this, the implementation of a combined severity and needs based approach replacing traditional risk stratification at Gold Coast Mental Health and Specialist Services was associated with a 35% reduction in suicide attempt re-presentations (62).

**Figure 1:**
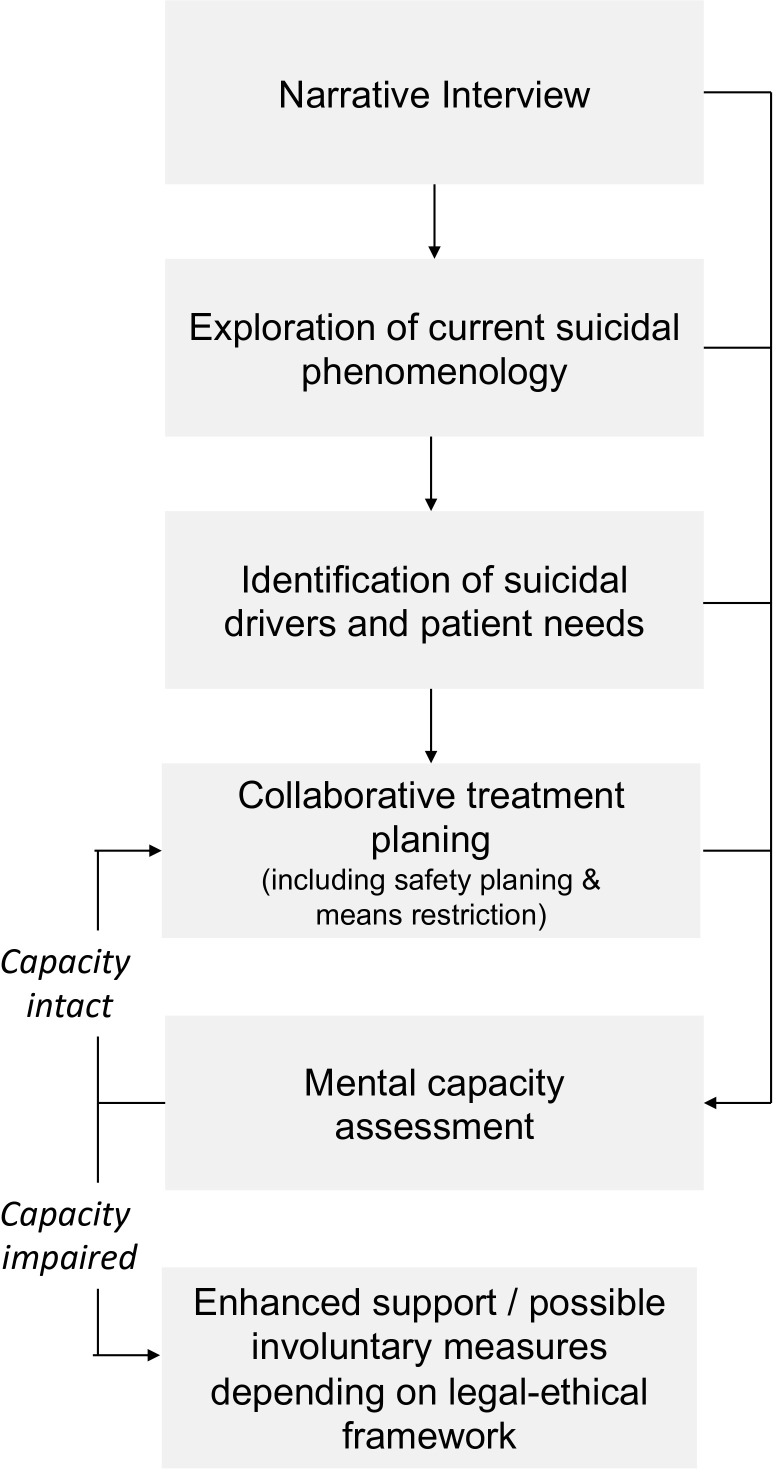
Integrated clinical decision algorithm for suicidality assessment: a needs-, severity-, and capacity-informed approach.

Should it become evident in the course of such a suicide-focused conversation that the patient lacks decision-making capacity (57), the collaborative, needs-based framework necessarily gives way to a more protective capacity-based approach, with the clinician ensuring transfer to a safe setting. Importantly, this decision is not grounded in the assumption that the clinician can determine an elevated risk of suicide with certainty, but rather in the judgment that, in the patient’s current state, they are unable or should not be expected to make autonomous decisions. **Figure 1** illustrates the proposed sequence of the comprehensive approach.

## 4 Implications for risk assessment in time- and resource-limited settings

In settings where there is neither the time nor the resources for a detailed collaborative, needs-based assessment of suicidal ideation and behavior (e.g., prisons, schools), screening questionnaires or checklists are often used to identify individuals at risk of suicide. Based on the empirical findings presented, screening instruments in prisons, schools, emergency rooms, and other institutional settings carry a fundamental risk. Rather than improving safety for those affected, they may create a false sense of security, giving practitioners and institutions a feeling of control that the evidence does not support (50).

It must be assumed that many people for example in prisons (63) and in facilities for refugees (64) belong to a risk population. Against this background, it can be argued that selective prevention strategies - or, in school contexts, universal prevention approaches [e.g., (65–67)], - may offer advantages over purely indicated prevention models. Selective suicide prevention in at-risk populations would imply that all individuals are regularly given opportunities to discuss suicidal ideation and behavior, receive psychoeducational information on suicidality and coping strategies, and are offered access to psychosocial, psychotherapeutic, or psychiatric support as needed. Importantly, such approaches also aim to create conditions that facilitate disclosure of suicidal ideation by engaging individuals in a manner that reduces barriers to communication (33). When suicidal ideation or behavior is disclosed, responses should be supportive and exploratory, as outlined above. In primary care contexts, if a general practitioner (GP) suspects that a patient may be experiencing suicidal ideation, a collaborative and needs-based approach would involve asking about suicidal thoughts [e.g., (68)], providing a safe space for the patient to describe their experiences, and jointly exploring the types of support the patient considers appropriate to enhance his/her safety (69–71). Only in situations where there are substantial indications of impaired decision-making capacity would involuntary pathways - such as psychiatric consultation or hospital admission - be considered. Similarly, in emergency department settings following a suicide attempt, indicated preventive measures would generally be offered, ideally including a structured exploration of preceding suicidal experiences and the development of a collaboratively constructed safety plan (including means restriction counselling and information on emergency contacts). Initial studies have provided clear evidence that the risk of repeat suicide attempts can be effectively reduced through brief safety planning interventions (72) – even in emergency departments (73). Where safety planning is not feasible, at minimum, means restriction counselling should be provided. Importantly, such interventions should be delivered in a collaborative rather than coercive manner, as non-engaged or unilateral restriction of means may risk undermining the therapeutic alliance that is central to effective crisis care [cf. (74)].

## 5 Conclusion

The present paper has several limitations that warrant consideration. Specifically, the analysis is based on a narrative and theoretical synthesis rather than a formal systematic review, the proposed clinical alternatives are supported by varying levels of empirical evidence, and the associated legal implications may differ across jurisdictions. Nonetheless, the evidence reviewed in this paper leads to a clear conclusion: suicide risk assessment, in its current form, does not allow for the reliable prediction of suicidal behavior. This is not a new insight and already has been documented since the 1950s, confirmed across meta-analyses spanning individual risk factors, clinical judgment, theoretical models, and artificial intelligence, and is now reflected in major clinical guidelines in multiple countries. Acknowledging this does not mean abandoning the assessment of suicidality. It means redefining its purpose. The goal of a suicide-focused conversation is not to classify risk but to understand the person. What led to the crisis? What maintains it? What does the patient need? These questions form the basis for case conceptualization, safety planning, and treatment.

This shift in orientation has practical consequences at every level of care. In individual clinical encounters, it supports collaborative and needs-oriented treatment planning. In institutional settings, it points toward selective prevention approaches that reach entire at-risk populations rather than attempting to identify individuals through screening. Legally, it is also well-supported: courts assess clinical decisions on what was knowable at the time, and disciplinary data show that complaints arise from insufficient therapeutic engagement, not from failing to predict the unpredictable.

Suicide cannot currently be predicted with sufficient individual-level accuracy to justify global risk stratification or high-stakes disposition decisions. It can, however, be approached with understanding, collaboration, and care to enhance safety for the patient.

## Data Availability

The original contributions presented in the study are included in the article/supplementary material. Further inquiries can be directed to the corresponding author.
